# Robust estimation of dementia prevalence from two-phase surveys with non-responders via propensity score stratification

**DOI:** 10.1186/s12874-023-01954-0

**Published:** 2023-05-27

**Authors:** Chong Shen, Minyue Pei, Xiaoxiao Wang, Yiming Zhao, Luning Wang, Jiping Tan, Ke Deng, Nan Li

**Affiliations:** 1 Center for Statistical Science, Department of Industrial Engineering, Tsinghua University, No. 30, Shuangqing Road, Haidian District, Beijing, 100084 People’s Republic of China; 2 Research Center of Clinical Epidemiology, Peking University Third Hospital, No. 49, Huayuan North Road, Haidian District, Beijing, 100191 People’s Republic of China; 3 Key Laboratory of Epidemiology of Major Diseases (Peking University), Ministry of Education, Beijing, People’s Republic of China; 4 Geriatric Neurology Department of The Second Medical Center & National Clinical Research Center for Geriatric Diseases, Chinese PLA General Hospital, No. 28, Fuxing Road, Haidian District, Beijing, 100039 People’s Republic of China

**Keywords:** Prevalence estimation, Missing data, Propensity score

## Abstract

**Background:**

Missing diagnoses are common in cross-sectional studies of dementia, and this missingness is usually related to whether the respondent has dementia or not. Failure to properly address this issue can lead to underestimation of prevalence. To obtain accurate prevalence estimates, we propose different estimation methods within the framework of propensity score stratification (PSS), which can significantly reduce the negative impact of non-response on prevalence estimates.

**Methods:**

To obtain accurate estimates of dementia prevalence, we calculated the propensity score (PS) of each participant to be a non-responder using logistic regression with demographic information, cognitive tests and physical function variables as covariates. We then divided all participants into five equal-sized strata based on their PS. The stratum-specific prevalence of dementia was estimated using simple estimation (SE), regression estimation (RE), and regression estimation with multiple imputation (REMI). These stratum-specific estimates were integrated to obtain an overall estimate of dementia prevalence.

**Results:**

The estimated prevalence of dementia using SE, RE, and REMI with PSS was 12.24%, 12.28%, and 12.20%, respectively. These estimates showed higher consistency than the estimates obtained without PSS, which were 11.64%, 12.33%, and 11.98%, respectively. Furthermore, considering only the observed diagnoses, the prevalence in the same group was found to be 9.95%, which is significantly lower than the prevalence estimated by our proposed method. This suggested that prevalence estimates obtained without properly accounting for missing data might underestimate the true prevalence.

**Conclusion:**

Estimating the prevalence of dementia using the PSS provides a more robust and less biased estimate.

**Supplementary Information:**

The online version contains supplementary material available at 10.1186/s12874-023-01954-0.

## Background

Dementia is a neurodegenerative disease that leads to irreversible memory loss, language dysfunction, and difficulties in carrying out daily activities [1]. In an aging society, dementia has become a major public health challenge, affecting nearly 50 million people worldwide [2, 3]. This places a heavy burden not only on people with dementia and their families, but also on society and the economy. The total cost of caring for people with dementia is one of the largest healthcare expenditures for society, and is expected to reach $355 billion globally in 2021 [4]. China is also expected to spend $507.49 billion on dementia care in 2030 [5]. In addition, mild cognitive impairment (MCI), the intermediate state between normal and dementia, has also been the focus of research [1]. The number of people with dementia or MCI is increasing over time, leading to a growing healthcare burden in the future [6]. Therefore, an accurate prevalence estimation is essential to understand the disease, accurately assess the burden of disease, and make informed health policy decisions [6, 7].

Dementia research typically follows a two-phase survey approach [8]. In phase I, screening is carried out in the general population to identify high-risk participants, who then undergo systemic neuropsychological testing in phase II to obtain accurate diagnoses. However, there are significant challenges in conducting testing in phase II [9, 10]. Some of the essential tests are complex and can be difficult for participants with poor physical, hospitalization, and cognitive conditions to complete, leading to a high non-response rate in phase II [11]. This results in non-random missingness of final diagnoses and challenge to the estimation of dementia prevalence [12].

Missing data is a common issue in cross-sectional studies of dementia. Wu’s meta-analysis showed that large-scale prevalence studies in China had a response rate of around 90% [13]. However, traditional methods of dealing with missing data involve simply removing non-responders' data points from the analysis, which can lead to an underestimation of prevalence. This is particularly true for studies of dementia, as missing diagnoses in phase II are more likely to be associated with higher disease rates. In these cases, the missing mechanism is classified as missing not at random (MNAR) [14].

Unfortunately, we have found that many previous studies suffer from the defect of not properly dealing with missing data, resulting in underestimates of the prevalence and burden of dementia [15, 16]. For example, the Global Burden of Diseases, Injuries, and Risk Factors Study (GBD) calculated global prevalence through systematic reviews without correcting the potential bias due to missing data [4, 6]. In recent years, many researchers have made great efforts to address this critical issue. Tan et al. [17] proposed to impute the missing diagnoses in phase II via various imputation methods and compared the resulting estimated prevalence. There are some previous studies that have dealt with missing data in different ways in longitudinal studies [18–20], but not in cross-sectional studies. Chen developed a method called the Diggle-Kenward (DK) selection model to deal with MNAR [21]. However, there is still a lack of a logical strategy with an explicit protocol to properly handle the unobserved diagnoses that are MNAR in the field of dementia research.

In recent years, propensity scores (PS) have gained popularity in observational studies. Defined as the conditional probability of a unit being assigned to a particular treatment group given a set of observed covariates, PS can help balancing confounding factors across treatment or exposure groups [22]. We assume that when the observed indicators are included to build non-response PS model, the composition of independent variables in the model help us to diagnose the existence of MNAR. It will indicate MNAR, if the meaningful independent variables contain those associated with the outcome. In such cases, direct estimation, also known as marginal effects, may be influenced by several confounding factors.

In this study, we have addressed the challenge of handling missing data under the MNAR mechanism by introducing a general strategy using propensity score stratification (PSS) [22]. Within the stratification, the observed and missing data can be approximated as identically distributed, making it a conditional effects method. By applying the proposed strategy to estimate the prevalence of dementia among male veterans enrolled in the Chinese Veteran Clinical Research (CVCR) platform, we obtained an unbiased prevalence estimator that is robust to different imputation methods.

## Methods

### Data source

This study was based on a sub-project of the Chinese Veteran Clinical Research (CVCR) Platform for the Assessment of Non-Communicable Diseases program. It was a multicenter, two-phase, cross-sectional study to estimate the prevalence of dementia and MCI. The design and protocol of the CVCR have been approved by the Ethics Committee of the Chinese People's Liberation Army (PLA) General Hospital(No. 20090820–02) [23]. In this study, we reviewed the de-identified database of the CVCR platform, and the design and protocol of data analysis were approved by Ethics Committee of the Peking University Third Hospital (No. M2016114 and M2017055).

### Study population

**Fig. 1:**
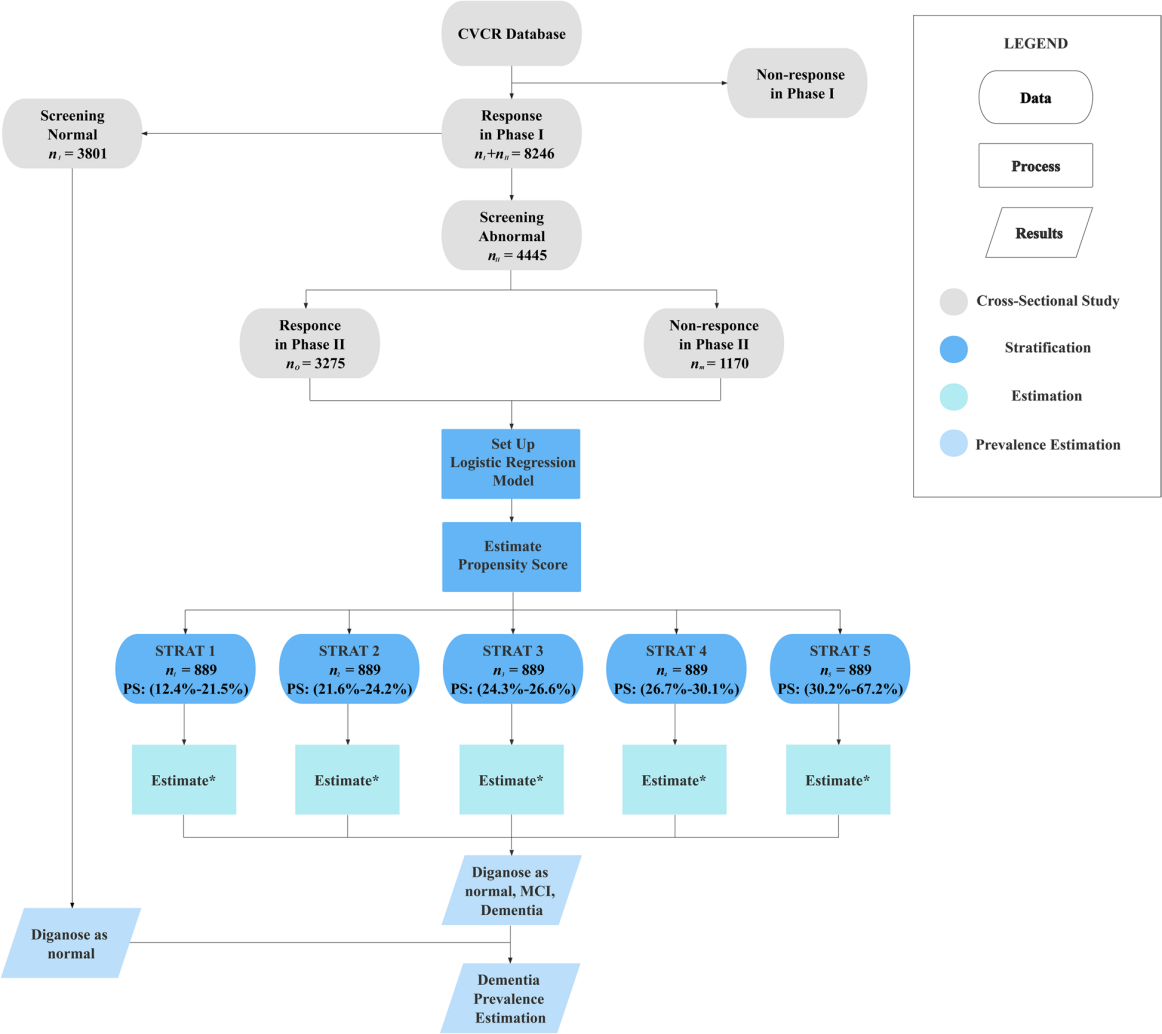
Schematic diagram of imputation and propensity score in each stratum * Estimation methods included simple estimation, regression estimation, and regression estimation with multiple imputation. Each stratum was imputed in the same way and the estimations were then combined. The covariates used by regression estimation were different at each stratum. Note: Propensity score for non-response was calculated by logistic regression

The CVCR survey included 8,246 veterans aged 60 and over from 277 veteran communities in 18 cities who had registered on the CVCR platform and had been living continuously in a veteran community for at least one month. Of the 8,246 veterans who participated in the survey, ${\mathrm{n}}_{\mathrm{I}}=\mathrm{3,801}$ were diagnosed as normal in phase I, and only the remaining ${\mathrm{n}}_{\mathrm{II}}=\mathrm{4,445}$ were enrolled in phase II. Among the participants enrolled in phase II, 1,170 failed to complete enough neuropsychological batteries to obtain a clinical diagnosis and were defined as non-responders, while the others received a specific diagnosis (589 normal, 1979 MCI, 707 dementia). Figure 1 illustrates the main results in both phases of the survey, and detailed baseline characteristics of the participants are shown in Table 1.

**Table 1:** Baseline characteristics of the sub-population with and without diagnosis in phase II

	**Sub-population with diagnosis (*n* = 3275)**	**Sub-population without diagnosis (*n* = 1170)**	***P*-value**	**SMD**
Age,	$82.89\pm 3.72$	$83.08\pm 3.76$	$0.14$	$0.05$
Year of Education	$7.52\pm 4.58$	$7.55\pm 4.76$	$0.86$	$0.01$
PADL score	$12.25\pm 5.01$	$13.01\pm 6.00$	$<0.001$	0.14
IADL score	$15.28\pm 8.74$	$15.86\pm 9.29$	$0.06$	0.06
MMSE score	$24.91\pm 5.16$	$24.15\pm 5.02$	$<0.001$	0.15
MoCA score	$21.48\pm 5.56$	$20.00\pm 5.21$	$<0.001$	0.27

Data are expressed as mean ± SD

*Abbreviations*: *SMD* standardized mean difference, *MoCA* Montreal Cognitive Assessment, *MMSE* Mini-Mental State Examination, *PADL* physical activities of daily living, *IADL* Instrumental activities of daily living. *P*-values were calculated using t-test or Wilcoxon signed-rank test, depending on the distribution of covariates. A two-tailed *P*-value < 0.05 was considered statistically significant. The PADL score, MMSE score, and MoCA scores of the two sub-populations were significantly different in the sense that the *P*-value was less than 0.05, a result that still holds after Bonferroni correction

### Diagnostic criteria

The Montreal Cognitive Assessment (MoCA), the Mini-Mental State Examination (MMSE) and the Activities of Daily Living (ADL) scale were used in the phase I to assess participants' cognitive and physical conditions [24]. Socio-demographic data (age, gender, education and living conditions) were collected by the investigators in a face-to-face interview.

In phase II, systemic neuropsychological tests were used to assess memory, language, visuospatial perception, calculation, abstract reasoning and executive function. Clinical diagnoses were made on the basis of a joint consideration of the patient's medical history, systematic neuropsychological tests, physical examinations in internal medicine and neurology, head CT or MRI, and blood tests. The diagnosis of dementia was based on the Diagnostic and Statistical Manual of Mental Disorders-IV (DSM-IV) [25]. The core clinical criteria was recommended by the International Aging and Alzheimer’s Disease Association to diagnose MCI [26].

### Statistical analysis

The characteristics of responders and non-responders in phase II were described by the mean (± standard deviation). The results obtained from these analyses, as shown in Table 1, reveal significant differences between responders and non-responders in phase II, suggesting that the corresponding missing mechanism is obviously missing not at random (MNAR). The following analysis consists of three parts: setting up the PS model and stratification, estimating stratum-specific prevalence, and summarising for final estimation. The analysis protocol is shown in Fig. 1 with the specific analysis process and formulas detailed in the supplementary material.

Non-random missing responses pose a critical challenge to prevalence estimation in practice and may lead to biased results if not handled properly. In this study, we address this issue by recommending the use of propensity score stratification (PSS) framework [22]. The propensity score (PS) was used to assess the missing probability of diagnostic results for those who entered phase II. Logistic regression is commonly used to calculate propensity scores (PSs), with the response variable being whether the participant has a missing diagnosis. The independent variables in the regression model can first include all variables related to dementia, such as socio-demographic information and cognitive screening scores, and then use stepwise regression to filter out the significant variables. We can then simply partition all participants into 5 equally sized strata based on the quintiles of the empirical distribution of the estimated propensity score. Rosenbaum and Rubin (1983) showed that such a strategy leads to simplified unit stratifications, within each of which the unobserved responses are approximately missing at random (MAR).

Based on the stratification approach, we can estimate the prevalence of each stratum using two different estimation methods: simple estimation (SE) and regression estimation (RE). SE directly estimates the prevalence of each stratum by calculating the percentage of dementia among the responders in the stratum. Since the prevalence estimator follows a binomial distribution, its variance can be easily estimated. On the other hand, RE utilizes an ordered logistic regression model between the covariates ${\varvec{X}}$ and the response ${\varvec{Y}}$ to provide a more statistically efficient prevalence estimation with a smaller estimation variance. In contrast to the calculation of propensity scores, the responses in RE are ordinal variables with three categories: normal, MCI and dementia. Similarly, we first consider the full set of covariates in each stratum and then filter out the significant variables by stepwise regression, with possible differences in the final covariates used in each stratum. In addition, we can combine multiple imputation, which is a Bayesian method for imputing missing data multiple times, with the aforementioned estimation methods. In this study, we utilized regression estimation with multiple imputation (REMI) to estimate the prevalence of each stratum [12]. Specifically, we followed the concept of regression estimation in modeling each stratum, but employed a Bayesian approach to impute missing values. As a result, after several imputations, different versions of the complete dataset can be obtained for each stratum. We then pool the prevalence estimates from each dataset to obtain a final stratum-specific prevalence.

Given the stratum-specific prevalence estimates, we integrate them into a proper estimate of overall prevalence via a weighted average, where the weight of each stratum is proportional to the sample size within it. The main advantage of such a stratification-based strategy is that, after stratification, the missing responses within each PSS can be treated approximately as MAR, making it logically sound and technically convenient for statistical inference on the unknown prevalence.

All analyses were performed using R (version 4.0.2). Details can be found in Supplementary Material.

## Results

### Propensity score stratification and baseline characteristics in each stratum

To establish a proper PSS for participants in phase II of the CVCR survey, we built a logistic regression model to describe how a participant's response rate varies with their covariates. The parameters of the model could be estimated using the observed indicators of responders and non-responders as responses. For each participant with a given set of covariates, their propensity score, derived from the fitted regression model, represents the predicted probability of them being a non-responder. Initially, we considered all recorded covariates that could potentially be associated with the absence of a diagnosis in the logistic regression, including socio-demographic information (e.g., age and year of education) and cognitive screening scores (e.g., MoCA, MMSE, and ADL). We gradually removed unimportant variables using stepwise regression with AIC as the model selection principle, resulting in a final model with only 4 covariates:

$$\mathrm{log}\left(\frac{PS}{1-PS}\right)=-0.56-0.08MoCA+0.04MMSE+0.03PADL-0.02IADL.$$

**Fig. 2:**
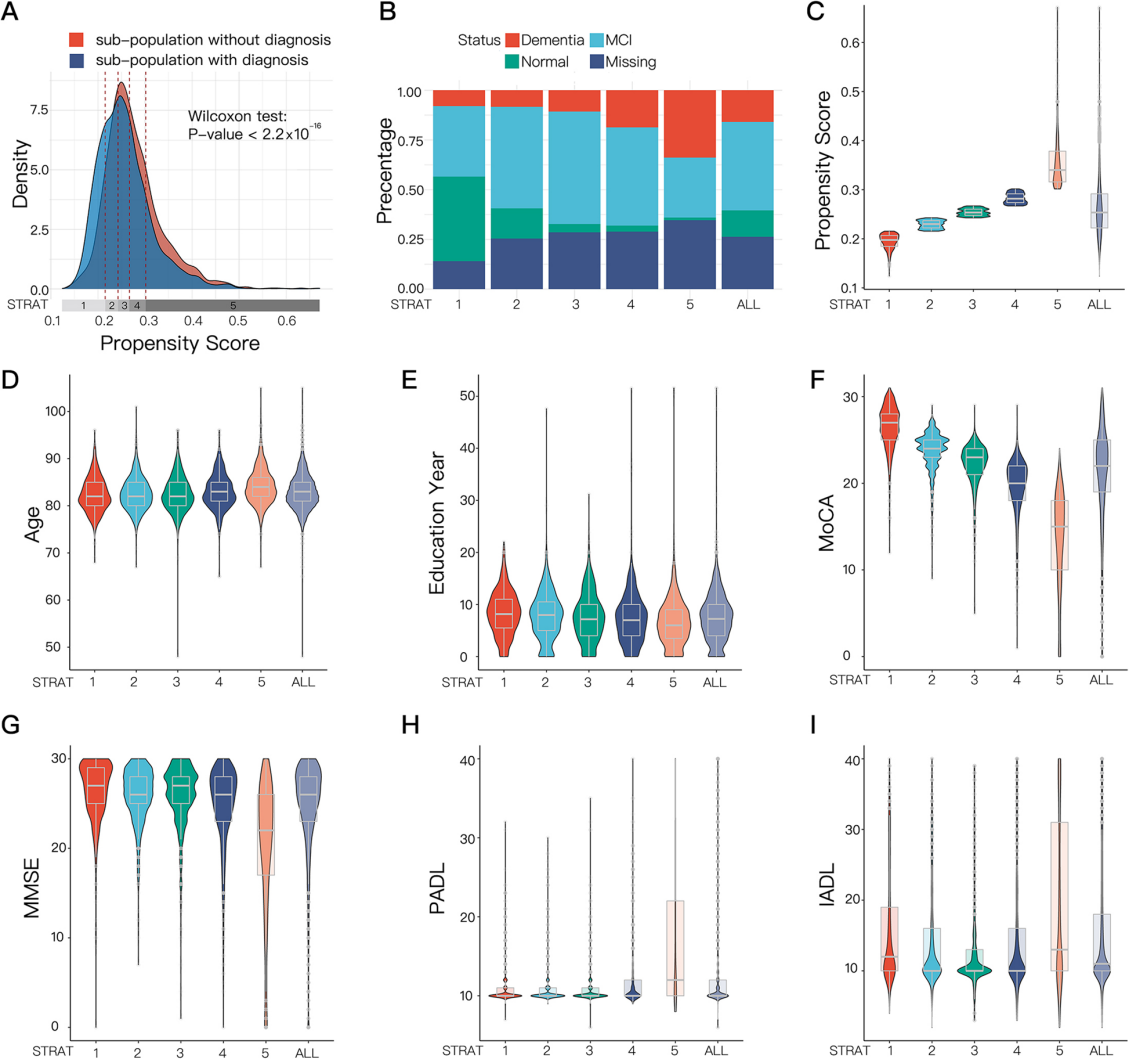
Distribution of PS on sub-populations with and without a diagnosis (**A**), and diagnosis status, propensity score and covariates in each stratum (**B**-**I**). **A** Four red dotted lines represent the quintiles of the propensity score. Wilcoxon signed-rank test showed that the PSs were on average higher in the sub-populations without diagnosis (*P*-value < 0.001). **B**-**I** The X-axis of each graph is stratum 1 to 5, and all the data. Fig **B** represents the percentage of diagnosis status: dementia, MCI, normal and missing. Fig **C**-**I** represent the distribution of PS score (**C**), age (**D**), year of education (**E**), PADL score (**F**), IADL score (**G**), MMSE score (**H**), MoCA score (**I**) in each stratum

Figure 2A shows the distributions of estimated PSs for both responders and non-responders, with non-responders on average had higher PSs than responders. A Wilcoxon test was performed to confirm that the difference between the two PS distributions was statistically significant ($$P$$-value $$<0.001)$$. It is important to note that if the missing mechanism was MAR, there will not be a meaningful PS model based on variables related to dementia itself. At the same time, the risk of non-response calculated by such a model could not effectively distinguish non-response from response patients. This finding is consistent with our prior knowledge that the missing mechanism in phase II of the CVCR survey involvesmissing not at random (MNAR), which supports the need for a propensity score stratification framework to adequately address missingness in dementia surveys.

To stratify all participants based on quintiles of estimated propensity scores, we divided the range of all estimated propensity scores into five strata, with the same number of participants in each stratum. The stratum boundaries are indicated by red dashed lines in Fig. 2A. The PSs of stratum 1 to 5 range from low to high, representing a low to high risk of missing a diagnosis. Importantly, there were enough responders and non-responders in all five strata to ensure that we had enough information to estimate the prevalence within each stratum.

We observed that the proportion of normal, MCI and dementia differed in each stratum (Fig. 2B). As the PS increased, the proportion of dementia and MCI in the observed data gradually increased. For example, the proportion of dementia in stratum 1 was 7.99%, compared with 33.85% in stratum 5. In addition, the proportion of non-responders also increased.

Figure 2 further shows the distributions of various covariates and estimated PSs in each of the 5 strata. It was clear that some covariates (such as MoCA score, MMSE score, etc.) had markedly different distributions across the strata. In particular, there was a clear linear trend between stratum and MoCA score ($P<2.2\times {10}^{-16}$), as shown in Fig. 2F.

### Prevalence estimation for strata and overall population

The prevalence of dementia and MCI estimated using SE, RE and REMI within each PSS is shown in Table 2. The estimation method was the same for both, except that the response variable ${\varvec{Y}}$ was changed from dementia to MCI. We found that the prevalence estimated by the three methods was very consistent within each PSS. For example, in stratum 5, with the highest percentage of missing diagnoses (34.63%), the estimated prevalence of dementia by PSS-SE, PSS-RE, and PSS-REMI was 51.86%, 51.97% and 51.63%, respectively. Furthermore, the sum of the prevalence of MCI and dementia gradually increased from stratum 1 to stratum 5. The proportion of the normal population estimated by the PSS-RE in each stratum was 46.68%, 19.24%, 5.85%, 4.39% and 2.02%, respectively. This suggests that the higher the propensity score of the respondent, the higher the risk of cognitive impairment. In addition, the prevalence of MCI first increased and then decreased, being highest in stratum 3. This might be because the proportion of respondents with dementia increased rapidly in stratum 4 and 5, as presented in Fig. 2B.

**Table 2:** Estimated percentage of participants with different cognitive status within each PSS, phase II and CVCR

		STRAT 1 (%)	STRAT 2 (%)	STRAT 3 (%)	STRAT 4 (%)	STRAT 5 (%)	phase II (%) (***n***_***II***_ = 4445)	CVCR (%) (***n***_***I***_** + *n***_***II***_ = 8246)
		(***n***_***1***_ = 889)	(***n***_***2***_ = 889)	(***n***_***3***_ = 889)	(***n***_***4***_ = 889)	(***n***_***5***_ = 889)		
PSS-SE	MCI	41.28 (0.62)	68.5 (0.79)	79.19 (0.73)	69.52 (0.83)	46.12 (0.98)	60.90 (0.36)	32.83 (0.19)
	dementia	9.34 (0.37)	11.14 (0.53)	14.96 (0.64)	26.32 (0.79)	51.86 (0.99)	22.70 (0.31)	12.24 (0.17)
PSS-RE	MCI	43.31 (0.61)	68.95 (0.73)	79.98 (0.65)	69.74 (0.75)	46.01 (0.80)	61.60 (0.32)	33.20 (0.17)
	dementia	10.01 (0.41)	11.81 (0.47)	14.17 (0.52)	25.87 (0.68)	51.97 (0.78)	22.79 (0.26)	12.28 (0.14)
PSS-REMI	MCI	43.19 (0.63)	69.18 (0.83)	79.64 (0.72)	69.97 (0.83)	46.34 (0.93)	61.69 (0.36)	33.25 (0.19)
	dementia	9.79 (0.41)	11.36 (0.52)	14.51 (0.61)	25.76 (0.78)	51.63 (0.91)	22.63 (0.30)	12.20 (0.16)

Data are expressed as mean (standard deviation) of the percentage

*Abbreviations*:* STRAT* stratum

**Fig. 3:**
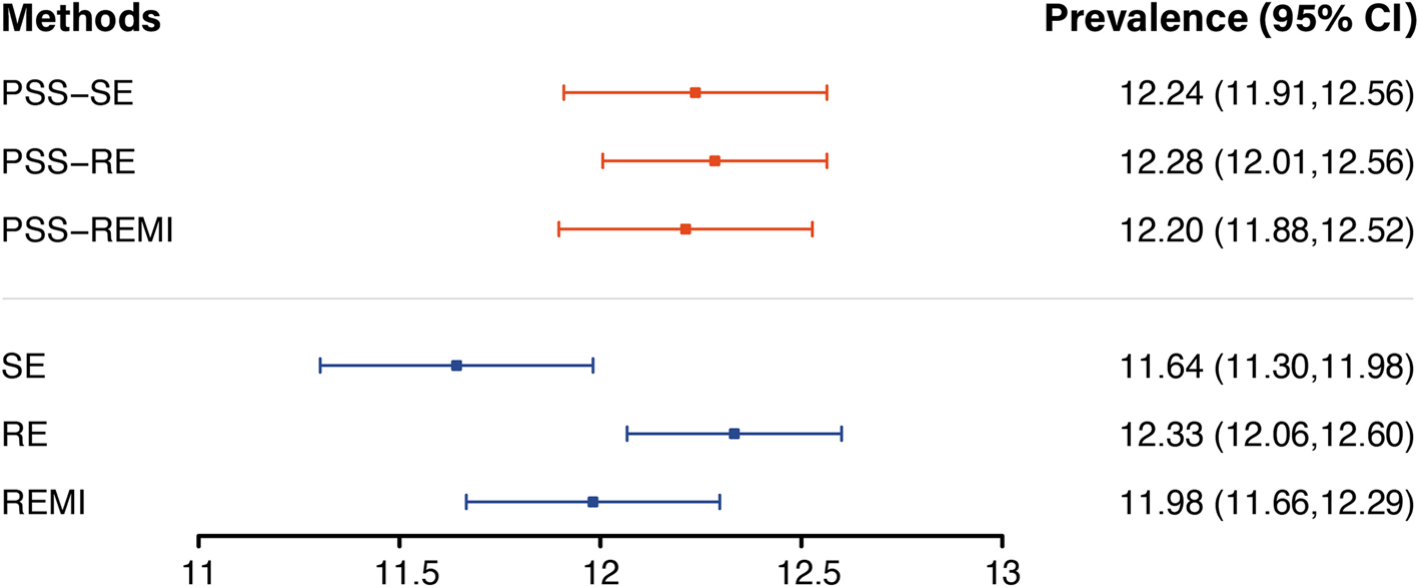
Comparison of estimated prevalence of CVCR between three methods with and without PSS. Estimated prevalence using different imputation methods are expressed as mean (95% CI). The upper panel represents the results with PSS

Figure 3 visualizes the estimated prevalence and corresponding 95% confidence intervals for the whole population (${n}_{I}+{n}_{II}$ participants) based on different estimation methods using PSS (red intervals), as well as the results without PSS (purple intervals). Under the PSS framework, the prevalence and corresponding intervals obtained by the three estimation methods are highly consistent, indicating that all three methods effectively impute missing data and estimate prevalence when the missing mechanism within each stratum is MAR. However, on an unstratified basis, where the essential requirement for dealing with missing data is not met, the estimated prevalence of the different methods differs significantly and the confidence intervals of the SE and RE do not even overlap. Furthermore, RE, which uses information on covariates, can partially counteract the effect of MNAR mechanisms in the data, leading to similar results as the PSS-based approach. However, it is important to emphasize that the direct use of RE is still very risky.

Compared with the prevalence of 9.95% before the treatment of missing data, the mean estimated prevalence under the PSS framework was 12.24%, suggesting that the true prevalence may have been underestimated in the past.

### Compared the prevalence with published data by 80–89 age male

**Fig. 4:**
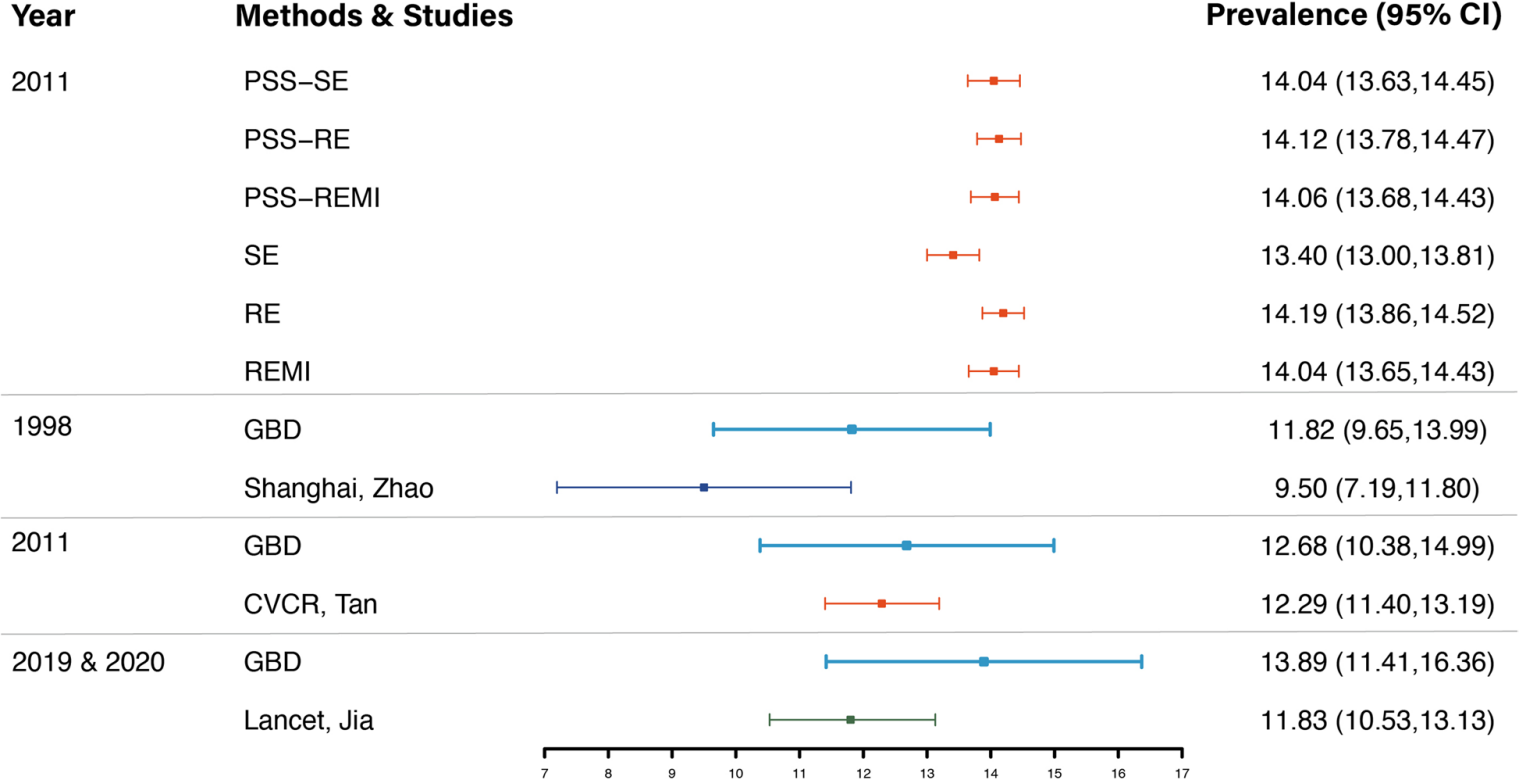
Prevalence of dementia in CVCR and other studies in China by male group aged 80–89. Different colours represent different research years, dark blue for 1998, orange for 2011 and green for 2019. The results of the GBD study are shown in light blue and were considered as a reference in different years. The top two panels show the estimated results in CVCRwith and without PSS. Prevalence is expressed as the mean (95% CI). Prevalence in 2011 was estimated from the CVCR platform, and prevalence in 1998 and 2020 was estimated from published data by Zhao et al. and Jia et al., respectively. GBD data were obtained from the GHDx database

To compare the prevalence estimation with other studies, we chose the published data from Zhao et al. [27] and Jia et al. [28], which were large cross-sectional studies in China and could better represent the prevalence of dementia in China. The prevalence and the confidence interval were calculated from published second-hand data. The results were presented as a forest plot (Fig. 4). As the final prevalence was standardized differently and the 80–89 year old group had less weight in published studies, which was not the case for the CVCR where this age group had a larger population, we chose the 80–89 year old male group to calculate the prevalence for better comparability.

When comparing the group of men aged 80–89 years, the estimated prevalence of dementia in the CVCR was higher than in other dementia studies, as shown in Fig. 4. For older individuals aged 80–89 years, the prevalence of dementia in this study (12.29%) was similar to the prevalence estimated by the GBD for China in the same year (12.68%). In other individual studies conducted in China during the same period, the prevalence was lower than the GBD estimates (9.50% vs 11.82% for 1998 [27], 11.83% vs 13.89% for 2019/2020 [28]). In general, the prevalence of dementia in older people is gradually increasing over time, as shown by the trend in the GBD study.

Using PSS, the prevalence estimated by the three methods was approximately 14% in the 80–89 year old male group, which would increase the estimated prevalence in the CVCR platform by approximately 1.7%. In contrast, missing data were not imputed in the studies by Zhao and Jia, and it is reasonable to assume that the true prevalence rate would be higher if missing values were imputed. Based on our projections, after imputing missing data using PSS, we predicted that the prevalence in 1998 and 2020 would increase to about 11.2% and 13.5%, respectively, according to the Zhao and Jia studies.

## Discussion

When we used different methods to estimate dementia diagnoses in patients who did not respond, the prevalence of dementia increased to 13.40–14.19% in the 80–89 age group. In a previous study [29], we compared the characteristics of responders and non-responders in phase II. It showed that non-responders in phase II were older, in poorer physical health, with lower cognitive performance, suggesting that non-responders were more likely to have dementia. Moreover, participants who screened positive for dementia in phase I had a higher rate of non-response. All this suggests that dementia and non-response in the dementia survey are closely related, and that MNAR is involved in the mechanism of missing dementia diagnosis [30]. Non-random non-response is a critical challenge for prevalence estimation in practice and can lead to biased results if not properly managed [31]. These may explain the underestimation of dementia prevalence when non-responders were ignored. When non-responders were ignored, the prevalence of dementia in male aged 80–89 years was lower in the CVCR than in the GBD (12.29% vs. 12.68%). After imputation for non-response, the prevalence of dementia in the CVCR was about 14%, suggesting that the true prevalence of dementia in this age group may be higher. Similarly, the prevalence of dementia in the population as a whole is likely to be higher than currently reported.

This study found that after using SE, RE and REMI to infer a diagnosis of dementia in non-responders, there were differences in prevalence in the population (11.64%, 12.33%, 11.98%). This is similar to the results of previous analyses based on the CVCR [17]. In previous studies, when the prevalence of dementia was estimated after imputing missing data using stratified weighting (SW), inverse probability weighting (IPW), hot-deck imputation (HDI) and ordinal logistic regression (OLR), the prevalence estimates ranged from 10 to 16% for dementia and showed greater variation. Some research has shown that by setting up a PS model, the association between missing propensity and dementia can be removed, thereby improving the performance of imputation models [32–35]. PSS would therefore be an ideal strategy. We recommend that this critical issue be addressed under the framework of the PSS: establish PS model and determine the missing mechanism, stratified veterans by the missing propensity score, then the same methods were used to estimate the prevalence of participants in each stratum, and finally the prevalence was pooled. After these procedures, the consistency of the estimated prevalence of the different methods was improved (12.24%, 12.28%, 12.20%). MoCA and MMSE scores reflecting cognitive function were used to stratify the probability of non-response. Therefore, the cognitive level of patients in each stratum is more similar. At this point, the absence of diagnosis was weakly correlated with cognitive function itself in each stratum, and the missing mechanism was closer to MAR. In this way, consistency between different methods can theoretically be improved. The results of this study also suggest that controlling for MNAR may be more important than models that impute missing data [31].

Three methods can be used to estimate the prevalence of each stratum: SE, RE and REMI. SE is based on the expectation and variance of the observed proportion of people with dementia to impute missingness, which does not rely on covariates and is sensitive to MNAR. In RE, participants in each stratum have different characteristics, so we choose different covariates to build the logistic model, and the same covariate will have different estimated coefficients in different stratum. As a method of imputation rather than estimation, multiple imputation must be combined with estimation methods such as simple estimation or regression estimation [36]. The results of multiple imputation will generally have a larger variance, which is consistent with its aim of taking full account of the uncertainty in the data.

However, in the published literature on the prevalence of dementia in China, estimates of prevalence in the 80–89 age group are imprecise. Some studies included fewer people in the population, which is reflected in the large confidence interval for prevalence estimates in this age group [27, 28]; other studies used only historical data to estimate the current prevalence in the 80–89 age group [8, 13]. This phenomenon widely existed in the research among the oldest-old around the world [2]. Missing data are inevitable not only in observational studies [37] but also in clinical trials [38]. Older non-responders are more likely to have the disease being studied. Therefore, an optimal method for dealing with missing data is needed [39]. With the development of an ageing society, there will be an increasing number of older people who are more likely to have dementia and other chronic diseases. And research on the elderly will have larger sample sizes and more variables, resulting in a more complicated missing mechanism [19], as more causes can lead to missing data. Traditional data imputation methods are not suitable, and a joint model in statistics will be a new direction for dealing with missing data in future medical research.

The study found that the prevalence of dementia may be underestimated by 2%. An accurate estimate of dementia prevalence could help guide policy and health care resources. A study conducted to simulate resource use for dementia in Australia found that age-related health resource use increased as the dementia population grew. The study also found that the lack of provision of residential aged care could put a strain on hospital resources. In addition, a study reported that neurological disorders are among the leading causes of disability and death, highlighting the need for more cost-effective and rational resource allocation. Accurate prevalence estimates can help to effectively address the challenges posed by pension shortages and an ageing population.

The findings of this study should be interpreted alongside its limitations. First, for stratification, dividing the population equally by the quintiles of the propensity score is a simple and effective method but it is not the only one. In more extreme scenarios, for example if there are not enough responders in a particular stratum, we can reduce the number of strata with some loss of precision or make the stratum include more people. In the estimating overall prevalence, the weighted average method we use will remove the effect of differences in sample size between strata. Also, in the PS model, only some of the collected covariates were considered, while there are still some confounders that cannot be assessed. In addition, our study only used data from one platform to build this model, which may limit the generalizability of the methods. Therefore, external validation is needed in further research.

## Conclusion

In conclusion, stratifying data according to the missing propensity and using appropriate prevalence estimation methods for each stratum can produce reliable estimates of the prevalence of dementia that are higher than the original estimates without accounting for missing data. Moreover, after PSS, the results of different estimation methods are more consistent.

## Supplementary Information

**Additional file 1.**

## Data Availability

All results presented in this study are objectively shown in this article and/or its Additional file. The R code of the study is available from corresponding authors Ke Deng and Nan Li. The datasets generated and/or analysed during the current study are not publicly available due to privacy or ethical restrictions but are available from corresponding author Jiping Tan on reasonable request.

## Abbreviations

CVCR: Chinese Veteran Clinical Research
PSS: Propensity score stratification
PS: Propensity score
SE: Simple estimation
RE: Regression estimation
REMI: Regression estimation with multiple imputation
GBD: Global Burden of Diseases, Injuries, and Risk Factors Study
MCI: Mild cognitive impairment
MNAR: Missing not at random
MAR: Missing at random
MoCA: Montreal Cognitive Assessment
MMSE: Mini-Mental State Examination
PADL: Physical activities of daily living
IADL: Instrumental activities of daily living
