# Supplementary material for: Robust estimation of dementia prevalence from two-phase surveys with non-responders via propensity score stratification
Source: BMC Med Res Methodol. 2023 May 27;23:130. doi: 10.1186/s12874-023-01954-0 (PMC10224322; doi:10.1186/s12874-023-01954-0)
Supplement: Supplementary file 1 — Additional file 1. [file 12874_2023_1954_MOESM1_ESM.docx]

**SUPPLEMENTAL MATERIAL CONTENTS**

**SUPPLEMENTAL METHODS**

*1 Propensity score stratification*

Proposed by Rosenbaum and Rubin (1983) [1], *propensity score* (PS) was defined as the conditional probability of a unit $i$ being assigned to a particular condition $Z_{i}$, e.g., treatment /control or observed/missing, given a set of observed covariates $\boldsymbol{X}_{\boldsymbol{i}}=(X_{i1},\cdots,X_{ip})$, i.e.,

$\pi_{i}=\Pr\left( Z_{i}=1 \mid\boldsymbol{X}_{i} \right),$

where $Z_{i}=1$ stands for the fact that the response $Y_{i}$ of unit $i$ is missing in our case, with $Z_{i}=0$ stands for the observed $Y_{i}$. In practice, logistic regression is often used to model the propensity score $\pi_{i}$, i.e.,

$$log\frac{\pi_{i}}{1-\pi_{i}}={\boldsymbol{\beta}^{\boldsymbol{T}}\boldsymbol{X}}_{\boldsymbol{i}}\boldsymbol{,}$$

where $\boldsymbol{\beta}=\left( \beta_{1},\cdots,\beta_{p} \right)^{T}$ are the regression coefficients that can be estimated by its maximum likelihood estimate (MLE) $\hat{\boldsymbol{\beta}}$from the observed data $\{\boldsymbol{X}_{i},Z_{i}{\}}_{i=1}^{n}$.

*1.1 Variable selection for establishing propensity score*

To establish the logistic regression model for estimating propensity score, we considered all recorded covariates potentially associated with the absence of diagnosis, including sociodemographic information (e.g., age and years of education) and cognitive screening scores (e.g., MoCA, MMSE and ADL). Backward stepwise selection was then used to choose variables, i.e., we began with a saturated model that contained all variables under consideration and then sequentially removed the least significant variables one after the other, until all remaining variables in the model were statistically significant. In practice, we could achieve this procedure conveniently by calling the "Stats" function in R package.

*1.2 Data stratification based on propensity score*

As pointed out by Rosenbaum and Rubin [1], the propensity score $\pi$ enjoys the attractive property that the condition variable $Z$ is independent of covariates $\boldsymbol{X}$ given $\pi$, i.e.,

$$\boldsymbol{X}\perp Z\mid\pi,$$

indicting that response $Y$ is missing completely at random within the stratification of units with the same propensity score $\pi$. More importantly, the unit stratification based on propensity score $\pi$ is the optimal one among all possible stratifications, in sense that it is the coarsest stratification satisfying the above conditional independence condition.

In practice, we can simply partition all involved units into 5 stratifications of equal sizes based on the quantiles of the empirical distribution of the estimated propensity score $\hat{\pi}$ derived based on $\hat{\boldsymbol{\beta}}$. Rosenbaum and Rubin [2] showed that such a strategy leads to simplified unit stratifications within which the MAR condition approximately holds, and performs reasonably well for most practical problems.

*2 Prevalence estimation* *for the entire population via propensity score stratification*

For a prevalence estimation problem with missing responses that are missing not at random, establishing *propensity score stratifications* (PSS) would be an ideal strategy to overcome the conceptual challenges due to missing data. Because the responses are approximately missing at random within each PSS, we can get unbiased estimate of the prevalence within each PSS conveniently, and then integrate them into a proper estimate of the overall prevalence via weighted average.

To be concrete, let $\mathcal{S}_{1},\cdots,\mathcal{S}_{5}$ be the established PSSs, $n_{i}$ and $p_{i}$ be the number of individuals and the prevalence of dementia in $\mathcal{S}_{i}$ respectively. Apparently, we have $n_{\mathrm{II}}=\sum_{i}^{5} n_{i}$. Based on the weighted average formula, it is straightforward to see that the overall prevalence $p$ can be expressed as a function of the stratum-spceifc prevelances, i.e., $p_{1},\cdots,p_{5}$, as below:

$$p=\frac{\sum_{i}^{m} n_{i}p_{i}}{n_{I}+n_{II}},$$

where $n_{I}$ denote the number of individuals diagnosed as normal in phase I. Assume that for each PSS $\mathcal{S}_{i}$, we already have $\hat{p}_{i}$ as an unbiased estimator of $p_{i}$, with ${V(\hat{p}}_{i})$ as the estimation variance, which can be estimated by ${\hat{V}(\hat{p}}_{i})$. Replacing $p_{i}$ by $\hat{p}_{i}$, we have the following estimate of $p$:

$$\hat{p}=\frac{\sum_{i}^{m} n_{i}\hat{p}_{i}}{n_{I}+n_{II}}.$$

Apparently,

$$E\left( \hat{p} \right)=p and V\left( \hat{p} \right)=\frac{\sum_{i}^{m} n_{i}^{2}V(\hat{p}_{i})}{\left( n_{I}+n_{II} \right)^{2}},$$

i.e., $\hat{p}$ is an unbiased estimator of $p$ with $V\left( \hat{p} \right)$ as the estimation variance. Similarly, subsititute $V\left( \hat{p}_{i} \right)$ with $\hat{V}\left( \hat{p}_{i} \right)$, we can estimate the estimation variance $V\left( \hat{p} \right)$ by

$$\hat{V}\left( \hat{p} \right)=\frac{\sum_{i}^{m} n_{i}^{2}\hat{V}(\hat{p}_{i})}{\left( n_{I}+n_{II} \right)^{2}}.$$

If we want to estimate the prevalence and variance of phase II, we can simply replace the $n_{I}+n_{II}$ in $\hat{p}$ and $\hat{V}\left( \hat{p} \right)$ above with $n_{II}$. Because the missing mechanism within each PSS $\mathcal{S}_{i}$ is roughly missing at random after stratification, different methods can be utilized to construct the unbiased estimation $\hat{p}_{i}$ and the corresponding variance estimation ${\hat{V}(\hat{p}}_{i})$ conveniently based on the observed data within PSS $\mathcal{S}_{i}$ (will be detailed in the next subsection). Therefore, such an estimation strategy based on PSS is logically sound and technically convenient. All efforts of prevalence estimating without PSS, however, always face critical logic barriers due to MNAR, and may lead to biased estimation that is sensitive to the estimation approach utilized.

*3 Prevalence estimation within each propensity score stratification*

Because the missing mechanism of each $\mathcal{S}_{i}$ can be roughly treated as missing at random, both simple estimation and regression estimation are ready to use for prevalence estimation within each PSS. Simple estimation directly estimates $p_{i}$ with the percentage of dementia patients among the responders in $\mathcal{S}_{i}$, without considering the information carried by covariates $\boldsymbol{X}$. Regression estimation, however, pursuits a more efficient estimation with smaller estimation variance via a linear regression model between covariates $\boldsymbol{X}$ and response $\boldsymbol{Y}$ to utilize the prediction power of $\boldsymbol{X}$ for $\boldsymbol{Y}$.

*3.1 Simple estimation*

Let $n_{io}$ and $n_{im}$ be the number of responders and non-responders in $\mathcal{S}_{i}$ respectively, and $m_{io}$ and $m_{im}$ be the number of dementia patients in the responders and non-responders in $\mathcal{S}_{i}$ respectively. Apparently, $m_{im}$ cannot be deirectly observed due to the missing resposnses of the non-responders. Simple estimation estimates $p_{i}$ by

$$\hat{p}_{i,SE}=\frac{m_{io}}{n_{io}},$$

whose mean and variance are approximately $p_{i}$ and $p_{i}\left( 1-p_{i} \right),$ because the $n_{io}$responders in $\mathcal{S}_{i}$ can roughly be treated as a group of simple random samples from all individuals in $\mathcal{S}_{i}$. Therefore, we can estimate $V\left( \hat{p}_{i,SE} \right)$, the estimation variance of $\hat{p}_{i,SE}$, by

$$\hat{V}\left( \hat{p}_{i,SE} \right)=\hat{p}_{i,SE}\left( 1-\hat{p}_{i,SE} \right)=\frac{m_{io}(n_{io}-m_{io})}{n_{io}^{2}}.$$

*3.2 Regression estimation*

As the response is ordinal variable with three categories: normal, MCI and dementia, we use ordered logistic regression to model the data. Regression estimation requires a separate selection of covariates $\boldsymbol{X}_{i}=(X_{i1},\cdots,X_{ik})$ that has explanatory effect on response $\boldsymbol{Y}_{i}=(Y_{i1},\cdots,Y_{ik})$ in each $\mathcal{S}_{i}$. Because the characteristics within each $\mathcal{S}_{i}$ are different, the selected covariates via stepwise regression would be quite different. This further shows that if we use only one model to predict the entire data, a biased estimation would be what we have. Regression estimation estimates $p_{ij}$ by

$$\hat{p}_{ij,RE}=\frac{\exp\left( \boldsymbol{\beta}_{i}^{\boldsymbol{T}}\boldsymbol{X}_{ij}-\kappa_{i} \right)}{1+\exp\left( \boldsymbol{\beta}_{i}^{\boldsymbol{T}}\boldsymbol{X}_{ij}-\kappa_{i} \right)},j=1,\ldots,n_{i}$$

where $\boldsymbol{\beta}=\left( \beta_{1},\cdots,\beta_{k} \right)^{T}$ and $\kappa_{i}$ are the parameters that can be estimated by its MLE $\hat{\boldsymbol{\beta}}$and $\hat{\kappa_{i}}$ from the observed data $\{\boldsymbol{X}_{i},\boldsymbol{Y}_{i}{\}}_{\boldsymbol{i=1}}^{\boldsymbol{n}_{\boldsymbol{io}}}$.

Therefore, we can estimate $E\left( \hat{p}_{i,RE} \right)$ and $V\left( \hat{p}_{i,RE} \right)$, the estimation mean and variance of $\hat{p}_{i,RE}$, by

$$E\left( \hat{p}_{i,RE} \right)=\frac{\sum_{j}^{n_{i}} \hat{p}_{ij,RE}}{n_{i}}=\sum_{j}^{n_{i}} \frac{\exp\left( \boldsymbol{\beta}_{i}^{\boldsymbol{T}}\boldsymbol{X}_{ij}-\kappa_{i} \right)}{n_{i}\left[ 1+\exp\left( \boldsymbol{\beta}_{i}^{\boldsymbol{T}}\boldsymbol{X}_{ij}-\kappa_{i} \right) \right]}$$

$$V\left( \hat{p}_{i,RE} \right)=\frac{\sum_{j}^{n_{i}} Var(\hat{p}_{ij,RE})}{n_{i}^{2}}=\sum_{j}^{n_{i}} \frac{\exp\left( \boldsymbol{\beta}_{i}^{\boldsymbol{T}}\boldsymbol{X}_{ij}-\kappa_{i} \right)}{{n_{i}^{2}\left[ 1+\exp\left( \boldsymbol{\beta}_{i}^{\boldsymbol{T}}\boldsymbol{X}_{ij}-\kappa_{i} \right) \right]}^{2}}.$$

*3.3 Prevalence estimation via multiple imputation*

In addition, we can estimate the variance of the above methods using simulation instead of analytic solution. Multiple imputation (MI) is a good way to do that [3]. MI creates several complete versions of the data by imputing the missing diagnoses multiple times. Each of the complete datasets is then analyzed with standard statistical methods and the results are pooled for final inference using the Rubin’s combination rule. It provides a valid inference relative to simply imputing one complete data as it could properly account for uncertainty due to missing information. This can be done with R package of "mice" (<https://github.com/amices/mice>).

*4 Selected covariates and its coefficients in Regression Estimation with and without propensity score stratification*

| Covariate | PSS-RE | | | | | RE |
| --- | --- | --- | --- | --- | --- | --- |
|  | STRAT 1 | STRAT 2 | STRAT 3 | STRAT 4 | STRAT 5 |  |
| Age | - | - | 0.1919 | - | - | - |
| Year of Education | - | - | -0.0142 | -0.0942 | - | -0.0192 |
| PADL score | - | - | 0.0767 | - | - | 0.0345 |
| IADL score | - | 0.1418 | - | 0.1581 | 0.0562 | 0.0296 |
| MMSE score | -0.4136 | -0.3401 | - | -0.1811 | -0.2246 | -0.1330 |
| MoCA score | -0.1489 | - | -0.4311 | - | - | -0.2772 |

notes: In PSS-RE and RE, separate ordered logistic regression model was set in each stratum, and the variables were selected by stepwise regression with AIC.

**References:**

1. Rosenbaum, P.R. and D.B. Rubin, The central role of the propensity score in observational studies for causal effects. Biometrika, 1983. 70(1): p. 41-55.

2. Rosenbaum, P.R. and D.B. Rubin, Reducing Bias in Observational Studies Using Subclassification on the Propensity Score. Journal of the American Statistical Association, 1984. 79(387).

3. Campion, W.M. and D.B. Rubin, Multiple Imputation for Nonresponse in Surveys. Journal of Marketing Research, 1989. 26(4).
